# The Role of Nutrition in Applied Physiology

**DOI:** 10.3390/nu17071227

**Published:** 2025-03-31

**Authors:** Alvaro López-Samanes

**Affiliations:** GICAF Research Group, Department of Education Research Methods and Evaluation Faculty of Human and Social Sciences, Universidad Pontificia Comillas, 28049 Madrid, Spain; alsamanes@comillas.edu

Nutrition in applied and exercise physiology explores the relationship between nutrients and human body functions, focusing on the biochemical processes involved in the absorption, digestion, and utilization of food, nutrients, and other dietary compounds, as well as their impact on human performance [1]. While research on the effects of nutrition in this field (i.e., applied/exercise physiology) dates back to the 20th century [2], the number of publications has increased significantly, particularly since the 1960s, following pioneering studies by Scandinavian physiologists Bergström and Hultman, whose groundbreaking studies demonstrated that muscle glycogen is the primary carbohydrate substrate utilized during exercise in humans [3,4], a finding that likely contributed to the growing interest in the interaction between nutrition and applied/exercise physiology. Over the past 25 years (2000–2024), this interest has led to the publication of more than 1,700 scientific articles on the thematic area [5]; however, despite this remarkable growth, several gaps remain in nutrition in the applied and exercise physiology research area. Notably, there is a significant gender disparity, as only 0–8% of studies in this field have been realized with female participants exclusively [6,7]. Additionally, further research is needed to understand the impact of different dietary approaches (e.g., plant-based or ketogenic diets) on human performance [8,9], or to explore the potential role of sports nutrigenomics in overall health and performance [10]. To address these topics, we launched a Special Issue titled “*The Role of Nutrition in Applied Physiology*”, which attracted submissions from leading researchers and experts in the field. This Special Issue features seven high-quality original research articles and a narrative review. In this editorial, we provide an overview of the key findings from each manuscript.

**Dietary and Nutritional Supplements**

Regarding dietary supplements, only a few have been recognized by the International Olympic Committee as having strong evidence of enhancing performance when used in specific scenarios. These include caffeine, creatine, beta-alanine, sodium bicarbonate, and beetroot juice [11]. In this Special Issue, two of these supplements—creatine and caffeine—were explored. Gallo-Salazar et al. (2024) [Contribution 1] examined the effects of moderate caffeine ingestion (3 mg/kg body weight) on neuromuscular performance in well-trained hammer and discus throwers. Their study found that, compared to a placebo, caffeine increased throw distance, release speed, and modified throw performance, while no significant benefits were observed in jump height. Referring to side effects associated with caffeine ingestion, compared to the placebo, there was an increased prevalence of perceived activeness. Regarding creatine, a narrative review developed by Gutierrez-Hellín et al., (2024) [Contribution 2] explored its potential benefits beyond sports performance, particularly for women, vegans, and clinical populations. In women, creatine supplementation has been shown to enhance physical performance, muscle strength, and recovery, with minimal side effects such as temporary water retention. Vegans, who typically have lower dietary creatine intake, may experience improvements in both physical and cognitive performance with supplementation. In clinical populations, creatine supplementation appears to help reduce muscle loss in conditions such as sarcopenia, provide neuroprotection in neurodegenerative diseases, support cardiovascular health, and enhance energy metabolism in individuals with chronic fatigue syndrome and traumatic brain injuries. Overall, creatine monohydrate supplementation emerges as an effective strategy for improving muscle function, cognitive health, and recovery across diverse groups.

In addition to commonly studied supplements, a study by Ploszczyca et al. (2023) [Contribution 3] in this Special Issue examined the effects of D-Aspartic Acid (DAA) (i.e., an amino acid involved in hormone regulation, particularly in luteinizing hormone release and testosterone synthesis). The study investigated whether 6 g of DAA supplementation per day influenced hormonal (i.e., testosterone and cortisol) and hematological responses in male boxers undergoing 11 days of nocturnal normobaric hypoxia (simulating an altitude of 2500 m for 10–12 h per day). The results indicated that DAA supplementation had no significant effect on resting luteinizing hormone, testosterone, cortisol, or hematological parameters, including red blood cell count, hemoglobin, and hematocrit levels. The study concluded that short-term DAA supplementation at this dosage does not influence hormonal or hematological responses in athletes training under hypoxic conditions. Furthermore, dietary supplement use among elite athletes was also explored in this Special Issue. Sánchez-Oliver et al. (2024) [Contribution 4] analyzed the consumption of sports supplements among elite football referees in Spain, investigating differences based on competition level (First vs. Second Division) and referee type (main vs. assistant). Their findings revealed that 84.0% of referees reported using at least one dietary supplement, with significant differences in the use of medical supplements depending on the division and referee role. The most consumed supplements were whey protein, creatine, sports bars and drinks, and caffeine. The study concluded that dietary supplement use is highly prevalent among referees, with distinct patterns of consumption based on their competitive level and role.

**Dietary Patterns and Nutritional Interventions**

In recent years, there has been a growing interest in understanding dietary patterns across different populations, including both active and sedentary male adults. In this context, Johne et al. (2024) [Contribution 5] investigated the relationship between diet quality, serum brain-derived neurotrophic factor (BDNF) levels, and fatty acid profiles in physically active young men. Using cluster analysis, participants were categorized into three distinct dietary patterns, with Cluster 1 exhibiting the highest diet quality, characterized by a higher consumption of whole meal bread, milk, fruits, vegetables, and juices. Although no statistically significant differences in BDNF concentrations were found between clusters, a trend suggested that Cluster 1 had higher BDNF levels. Additionally, the study identified a negative correlation between BDNF levels and both body mass and BMI, indicating that body composition may influence neurotrophic factor levels. These findings suggest that diet quality plays a role in modulating BDNF levels, underscoring the complex interplay between nutrition, physical activity, and neurotrophic factors in maintaining optimal health.

Finally, the last two studies included in this Special Issue highlight the critical role of nutrition and physical activity in preventing health complications in adults or older adults. The first study by Cano-Lallave et al., (2024) [Contribution 6] examined the relationship between hepatic steatosis, adherence to the Mediterranean diet, and physical activity levels in individuals over 50 years old with a BMI over 25 kg/m^2^, revealing that low adherence to the Mediterranean diet and reduced physical activity were strongly associated with hepatic steatosis. Participants with hepatic steatosis showed poorer dietary habits and lower physical activity levels, emphasizing the importance of lifestyle modifications to prevent liver-related complications. Similarly, a study developed by Martin-Nieto et al. (2024) [Contribution 7] assessed nutritional status, vitamin D levels, and sarcopenia in older adults with hip fractures, finding that 79.7% of patients had vitamin D deficiency and malnutrition was prevalent, as indicated by low albumin and elevated blood urea nitrogen levels. Poor nutritional status, particularly low vitamin D and high BUN levels, was linked to increased mortality risk. Thus, both studies underscore the importance of diet quality and adequate nutritional assessment in older adults, as poor nutrition and physical inactivity contribute to metabolic disorders, musculoskeletal decline, and higher mortality risk, reinforcing the need for targeted interventions to improve long-term health outcomes.
